# Complex Learning in Bio-plausible Memristive Networks

**DOI:** 10.1038/srep10684

**Published:** 2015-06-19

**Authors:** Lei Deng, Guoqi Li, Ning Deng, Dong Wang, Ziyang Zhang, Wei He, Huanglong Li, Jing Pei, Luping Shi

**Affiliations:** 1 Center for Brain Inspired Computing Research (CBICR), Department of Precision Instrument, Tsinghua University, Beijing 100084, China; 2 Optical Memory National Engineering Research Center, Department of Precision Instrument, Tsinghua University, Beijing 100084, China; 3 Institute of Microelectronics, Tsinghua University, Beijing 100084, China; 4 Department of Engineering Product Design, Singapore University of Technology and Design, Singapore 138682

## Abstract

The emerging memristor-based neuromorphic engineering promises an efficient computing paradigm. However, the lack of both internal dynamics in the previous feedforward memristive networks and efficient learning algorithms in recurrent networks, fundamentally limits the learning ability of existing systems. In this work, we propose a framework to support complex learning functions by introducing dedicated learning algorithms to a bio-plausible recurrent memristive network with internal dynamics. We fabricate iron oxide memristor-based synapses, with well controllable plasticity and a wide dynamic range of excitatory/inhibitory connection weights, to build the network. To adaptively modify the synaptic weights, the comprehensive recursive least-squares (RLS) learning algorithm is introduced. Based on the proposed framework, the learning of various timing patterns and a complex spatiotemporal pattern of human motor is demonstrated. This work paves a new way to explore the brain-inspired complex learning in neuromorphic systems.

The emulation of complex spatiotemporal activities of the human brain cortex is central for understanding the learning behaviors, motor processing and cognition^1^. A variety of implementations based on both software and hardware are constantly developed for this purpose. Software-based artificial neural networks (ANNs) on von Neumann computer are widely applied to pattern classification, speech recognition, computer vision, and so on^2^,^3^. Whereas, software-based simulations occupy large area and consume high power^4^. In this context, various CMOS hardware-based neuromorphic systems have been developed^5^,^6^,^7^,^8^,^9^. However, the realization of complex learning in such systems is hindered by the lack of suitable device to model the synaptic plasticity^10^,^11^, until the recent invention of memristor^12^,^13^,^14^,^15^,^16^. Memristor, characterized by a pinched hysteresis loop of the I-V curve^17^, is a two-terminal nanodevice whose conductance can be gradually modulated under applied voltage or current^18^. This property is reminiscent of the synaptic plasticity^19^,^20^. In view of its attributes of low power, high speed and easiness to be crossbar integrated^21^,^22^,^23^,^24^,^25^, memristor has become one of the best candidates for artificial synapse in neuromorphic systems^19^,^26^,^27^,^28^,^29^,^30^,^31^,^32^,^33^,^34^,^35^.

Most of the previous memristive works are focused on feedforward architectures^20^,^36^,^37^,^38^,^39^, and a few cases with feedback connections^40^,^41^,^42^,^43^. Nevertheless, the feedforward networks lack internal network dynamics^1^ and the existing recurrent networks lack efficient learning algorithms^44^, which makes it difficult to support complex dynamics and emulate the learning functions of the brain. Inspired by the biological evidence that the complex learning ability arises from the efficient self-tuning of recurrent connections among neurons^45^, we propose a framework to support complex learning in memristive systems. The requirements of this framework are three-fold: (1) suitable device to model the synaptic plasticity; (2) bio-plausible recurrent neural network with ongoing internal dynamics; (3) efficient learning algorithm to adaptively modulate the synaptic weights.

To this end, we emulate the behaviors of excitatory and inhibitory synapses by combining two iron oxide-based memristors with one amplifier, and further build a bio-plausible recurrent memristive network^46^. The synaptic weights can be monotonously, consecutively and precisely modulated within a wide dynamic range. RLS algorithm, which is an efficient learning algorithm for training bio-plausible recurrent networks^1^,^46^, is applied to adaptively modify the synaptic weights. This makes it possible to realize complex learning functions in memristive networks. Various timing patterns are successfully learned, which demonstrates a powerful learning ability. In addition, the learning of a very complex spatiotemporal pattern of human motor is demonstrated in a large scale network, which is difficult to be realized in the previously reported memristive systems. This work suggests the possibility to explore the complex spatiotemporal learning behaviors in future large-scale neuromorphic computing systems.

## Results

### Complex learning framework in memristvie systems

**Figure 1:**
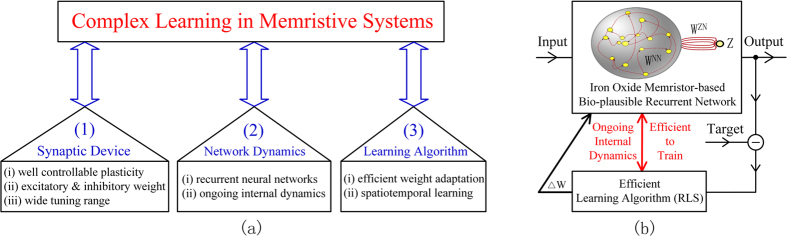
Framework for complex learning in memristive systems. (**a**) Framework for complex learning in memristive systems: (1) suitable synaptic device; (2) ongoing network dynamics; (3) efficient learning algorithm. (**b**) The proposed implementation including iron oxide memristor-based synapse, bio-plausible neural network and dedicated learning algorithm. The recurrent memristive network, constructed by iron oxide memristors with well controllable plasticity, is bio-plausible by eliminating the conventional strong feedback input. The RLS learning algorithm is particularly efficient in this recurrent network, which enables ongoing internal dynamics and efficient weight adaptation.

To support complex learning in memristive systems, three critical requirements must be fulfilled as shown in the framework in Fig. 1(a): (1) a suitable synaptic device (with well controllable plasticity, both excitatory and inhibitory synaptic weights, as well as a wide dynamic range of weight tuning); (2) ongoing internal dynamics (usually arises from recurrent neural networks); (3) an efficient learning algorithm for weight adaptation and spatiotemporal pattern learning. There do exist various memristive systems in the area of neuromorphic engineering. However, these systems fail to fulfil the three requirements simultaneously, and consequently have not shown the ability to support complex learning functions. For example, most of the systems are built on feedforward networks, which lack internal network dynamics^37^,^38^,^39^. Even if there exist memristive networks which are organized in a recurrent manner, the local learning rules such as the Hebbian rule and the spike timing dependent plasticity (STDP) rule^40^,^42^, or the typical delta rules^43^, are inadequate to train the recurrent neuromorphic networks^44^.

To address these issues, we propose an implementation of complex learning function to meet the above requirements. In the neural network model shown in Fig. 1(b), the internal yellow balls represent a group of neurons, which are recurrently connected by large amount of synapses denoted as red arrows. The readout unit ‘Z’ is connected to the internal neurons in a forward manner. In literature^46^, it is stated that the network structure in Fig. 1(b) is more biologically plausible, as it replaces the strong feedback input from an external pathway by adaptively modifying the internal synaptic weights. In order to achieve well controllable synaptic plasticity, we fabricate iron oxide memristors to construct the memristive network. The comprehensive RLS algorithm, as an efficient learning algorithm dedicated for the recurrent structure, is applied to adaptively modify the synaptic weights. This proposed memristive system is used to learn various complex spatiotemporal patterns. Considering the biological evidence that efficient modulation of recurrent synapses leads to complex neuronal activities^45^, the demonstrated results indicate the bio-plausibility of our implementation including the proposed synaptic device, network structure, internal dynamics, and learning algorithm.

### Tuning scheme for iron oxide memristor-based synapse

**Figure 2:**
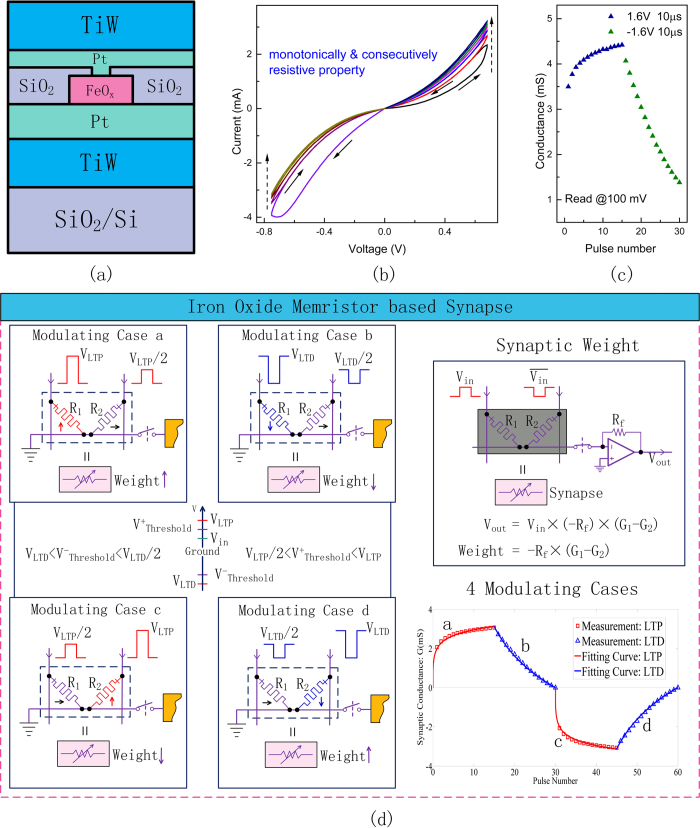
The iron oxide memristor based synapse and weight tuning scheme. (**a**) A schematic illustration of the iron oxide (FeOx)-based memristor device. (**b**) I-V curves of memristor under multiple triangle-shapes DC sweeps. A monotonous and consecutive distribution of resistance states during different sweeping cycles is observed. (**c**) The device conductance, related to the synaptic weight value, incrementally increases or decreases under consecutive positive pulse train or negative pulse train, respectively (+1.6 V for LTP and −1.6 V for LTD, each pulse train consists 15 pulses with identical duration of 10 us). (**d**) Two parallel memristors and one amplifier are combined to model a synapse. By introducing a reverse input voltage to invert the current, the two memristors make opposite contributions to the synaptic weight. Thus, the synapse can be modulated in four cases with different compositions of potentiating or depressing one of the two memristors. Only one memristor in each synapse is allowed to change its conductance during the modulation stage. An isolating switch connected to the dendrite wire of each neuron is used to isolate the memristive crossbar from the neuronal circuits when weight updating. The feedback resistance R_f_ of the inverter amplifier is used to adjust the dynamic range of synaptic weight.

The learning of complex spatiotemporal patterns in brain arises from adaptively modifying the synaptic weights^46^. Therefore, seeking a suitable device to simulate synaptic plasticity is the key to construct the bio-plausible network. Memristor is one of the most promising candidates^16^. In order to emulate the plastic behaviors of synapses, the conductance of memristor should be gradually tuned and well controlled. For this reason, we choose iron oxide^30^ as the resistance switching layer of memristor. As shown in Fig. 2(a), a 0.25 μm^2^-size memristor, consisting of a sandwich-like structure of TiW/Pt/FeO_x_/Pt/TiW, is fabricated. Figure 2(b) depicts the I-V characteristic curve in triangle-wave-shape DC voltage sweeping cycles, which is the iconic pinched hysteresis loops of memristor^17^. The hysteresis loops between each two adjacent sweeping cycles are almost ‘shoulder-to-shoulder’ but not overlapped. This indicates that the conductance of the memristor can be modulated consecutively and monotonically, which is suitable for synapse emulation.

We further investigate how to tune its conductance state under applied voltage pulse train. As shown in Fig. 2(c), the conductance of memristor either gradually increases or decreases by sequentially applying identical positive or negative pulses, respectively, which are considered as the long-term potentiation (LTP) and long-term depression (LTD) of synaptic plasticity^10^. In fact, the above conductance change only occurs when the amplitude of modulation pulses is above a threshold voltage and no significant conductance change is observed under applied voltages below the threshold^27^. This strongly nonlinear switching dynamics of iron oxide-based memristor makes it possible to work in two stages: (a) write stage when applying a relatively higher programming voltage, i.e., synaptic modulation stage; (b) nondisturbing read stage when applying a small voltage, i.e., neuronal processing stage^14^.

To build memristive networks, one of the key issues is how to realize both excitatory and inhibitory synapses. One solution is to leverage the joint conductance of two parallel memristors to model a synapse^39^,^47^. As illustrated in Fig. 2(d), the two memristors make opposite contributions when transferring V_in_ to V_out_. The left memristor (conductance G_1_) makes a positive contribution, while the right one (conductance G_2_) makes a negative contribution by reversing the input voltage to invert the current. Thus, we can increase the synaptic weight by potentiating the left memristor or depressing the right one; while decreasing the synaptic weight in the other way round. Therefore, the programming scheme is assigned into four modulating cases, as shown in Fig. 2(d). The entire system operates in two stages: the neuronal processing stage and the synaptic modulation stage. During the modulation stage, the isolating switch connected to the dendrite wire of each neuron is open and the memristive crossbar is isolated from the neuronal circuits; while after the weight updating, the switch is closed and the signal transduction from the synapses to the neuron is activated. It is worth mentioning that only one memristor in each synapse is allowed to change its conductance during the modulation stage. This constraint ensures a unique modulation path to drive the synaptic weight from any initial value to the desired one. In addition, when modifying one memristor within a synapse, we use the half-selected technique to avoid unintentional operation^39^.

The dynamic range of previous conductance-based synaptic weight^39^,^47^ is limited by the maximum resistance ratio of the memristive device. To address the issue, we design a structure to achieve a wide dynamic range of synaptic weight by combining two memristors with one amplifier. As shown in the top right of Fig. 2(d), the relationship between the input and output voltage is V_out_ = −R_f_ × (G_1_ − G_2_) × V_in_, where R_f_ is the feedback resistance of the inverting amplifier. Consequently, the effective synaptic weight is w = −R_f_ × (*G*_1_ − *G*_2_), which can achieve a relatively wide tuning range by increasing the resistance of R_f_. For example, as shown in Fig. 2(c), the minimum conductance value G_min_ and maximum conductance value G_max_ approximately equals to 1.5 mS and 4.5 mS, respectively. As expected, the joint conductance of two parallel memristors could be modulated gradually within the interval [–3 mS, 3 mS]. The modulation curve of each synapse is illustrated in the bottom right of Fig. 2(d). The simulation results are derived from the measurement data of single memristive device. Even though the resistance ratio is small (about 3), the weight value could be almost continuously modulated within a wide range [−1, 1], by fixing the R_f_ at 330 Ω.

### Complex learning under the proposed framework

**Figure 3:**
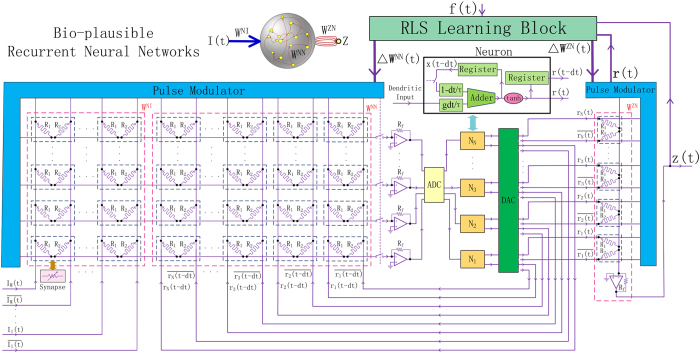
Circuit schematic of the memristive system for complex learning. The entire system is a mixed-analog-digital system, where the ADC block and DAC block are used to convert the signals. Each group of two parallel memristors in a small dotted box represents a synapse. The weight matrix **W**^NN^ is constructed by the memristive crossbar circuit and one dendritic amplifier on each neuron. The internal dynamics of neuron is implemented in the ‘Neuron’ block, which consists an accumulator and a hyperbolic tangent function blocks. The differential equation is solved numerically by transferring it to difference equation. The previous firing rate values are cached in registers and fed back into the network at next time step. Because of the fast switching of memristor, the RLS learning block and pulse modulator are adequate for the sequential operations of calculating the change of synaptic weight and modulating the corresponding synapse, respectively.

According to the requirements shown in Fig. 1(a), we construct a recurrent neural network based on the above synaptic device and introduce an efficient learning algorithm. The memristive network is shown in Fig. 3, where the activities of the internal neurons, e.g., membrane potential^48^ and the corresponding firing rate, are denoted as an N-dimensional vector of ***x*** and ***r***, respectively. The dynamics of recurrent network is governed by the following classical equations in neuroscience field^1^,^46^,^49^,^50^,^51^


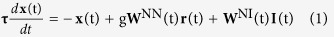



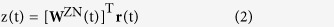



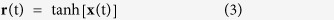


where **W**^NN^ denotes the N × N internal synaptic weight matrix, **W**^ZN^ denotes the weight vector from internal neurons to the readout unit, and z(t) denotes the output of readout unit which is a scalar value. The parameter g controls the dynamic regimes of the network by scaling the synaptic weight. The membrane time constant τ is usually of the order of several milliseconds to hundreds of milliseconds^11^,^52^. The external input vector and its connections to internal neurons, if available, are denoted as **I**(t) and **W**^NI^, respectively. The weight matrix **W**^NN^ could be either full or sparse. In particular, the sparse fashion is important for reducing hardware overhead in a large scale system and has been analyzed in Supplementary Fig. S6.

Based on the equations (1)–(3), 2, 3, we propose a hardware implementation of the recurrent memristive network. As shown in Fig. 3, the entire system is a mixed-analog-digital system, where the ADC block at the dendritic inputs and DAC block before the input voltage of the memristive crossbar are used to convert the signals. In the memristive crossbar, each group of two parallel memristors in a small dotted box represents a synapse. As a part of the synaptic device, the amplifier also has the ability to sum input currents from all parallel memristive branches. All the synapses connected to the same neuron share only one amplifier which serves as the integration function of dendrites. Thus, the synapse array is simplified to a memristive crossbar circuit and an amplifier column. In the ‘Neuron’ block, the differential equation is solved numerically by transferring it to difference equation. An accumulator^53^ is used to add up the previous membrane potential and current dendritic inputs in order to generate the current membrane potential. The previous activity states are cached in registers and fed back into the neural network at the next time step. The hyperbolic tangent function block could be implemented using CMOS technology^54^.

To implement complex learning under the proposed framework, an efficient learning algorithm at each time step is essential. Dedicated for the recurrent architecture, we introduce the recursive least-squares (RLS) algorithm^1^,^46^,^55^ into the system to adaptively modify the synaptic weights. One main capability of the brain is to learn complex spatiotemporal sequences, especially motor patterns in the prefrontal cortex^51^. Here, we demonstrate the learning and reproducing of a target pattern f(t) by minimizing the error between z(t) and f(t) through training the synaptic weights from random states. In fact, the learning process is to depress the chaotic dynamics and generate the regular target pattern by self-adapting the synapses, as shown in Supplementary Fig. S4.

According to the RLS algorithm, the error signal is defined as e(*t*) = z(*t*)−f(*t*), as shown in Fig. 3. At each time step, the synaptic weights on the dendritic tree of neuron i and the weight vector from internal neurons to readout unit are modified by


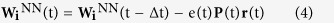



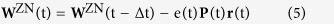


where **P**(*t*) is an N × N inverse correlation matrix, which is updated by


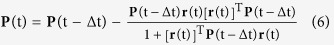


The **P**(t) matrix is initialized to **P**(0) = **I**/*α*, where **I** is the identity matrix. *α* is the learning rate parameter, which is reconfigurable according to specific target pattern. Usually, small *α* leads to fast learning but may result in divergence; while large *α* may lead to slow convergence.

In order to modify the weight value at each time step, we build a RLS learning block and a pulse modulator, which are responsible for sequentially calculating the weight increments according to Δ**W **= −e(t)**P**(t)**r**(t), and generating the pulse trains required to modulate the conductance according to the principle in Fig. 2(d), respectively. Ideally, the synapse should be gradually modulated to any weight from −1 to +1 by fitting appropriate R_f_. However, the conductance states of memristor are actually discrete. We propose a new method which leverages a differential pulse pair to modulate the conductance of memristor, so as to improve the modulation precision, as shown in Supplementary Fig. S2. By choosing suitable composition of potentiation and depression pulses, the relative accuracy could reach 0.3% that is adequate for the proposed system. Furthermore, considering the very slow firing rate of neuron (an average of 1−10 Hz)^56^, in contrast to the fast memristive switching (~ns − ~μs)^27^,^28^, we have enough time to precisely modulate the synaptic conductance to reach the desired value at each time step. We can further tune the weight value to achieve a higher precision by introducing the feedback modulating algorithm^39^,^57^ at the cost of longer modulation time. Therefore, in the sense of synaptic modulation precision, it is reasonable to use the continuous fitting curve to modulate the synaptic weights (Fig. 2 & Supplementary Fig. S1b) to support the simulation in this work^38^,^58^.

### Application for spatiotemporal pattern learning

**Figure 4:**
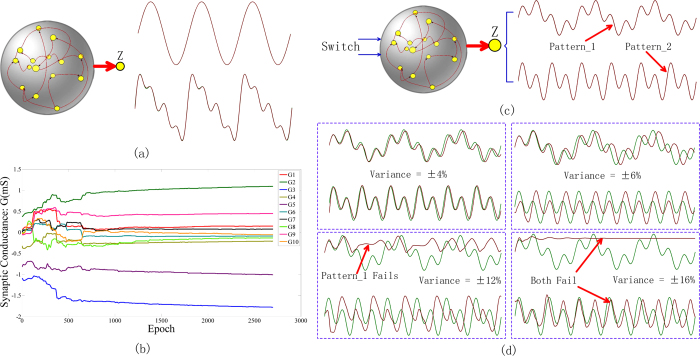
Examples of complex pattern learning and an analysis of the system immunity to device variance. (**a**) A variety of single target patterns are successfully generated, illustrated by a single sinusoid wave and a composite wave composed of four sinusoid functions. (**b**) Illustration of the joint conductance changing process of ten randomly chosen synapses when learning the single sinusoid wave. (**c**) Two target patterns of different compositions of sinusoid waves are generated in the meantime. The network has only one readout unit but two stable fixed points that could be switched by corresponding control inputs. (**d**) Analysis of the system immunity to device variance. The system reproduces the two target patterns well at a weight dispersion of ±6%, and reproduces one of the two target patterns even at ±12%. The system finally fails to track either of the two target patterns at ±16%.

Spatiotemporal activities learning are prevailing in human brain and is tempting for the applications of dynamical information processing. We employ the proposed memristive system to learn various spatiotemporal patterns, as illustrated in Fig. 4. In these examples, the target pattern f(t) plotted in green is learned and reproduced by the output z(t) in red. Figure 4(a) shows a periodic sinusoid wave pattern and a composite pattern composed of four sinusoid waves, where the actual output and the target pattern almost coincide, indicating that the error between the learned pattern and the target pattern is very small. A variety of more complex patterns are also successfully learned by this network, as presented in Supplementary Fig. S3.

Both the online training and offline training scheme are adopted to train the memristive network. As the online training is more technologically relevant, e.g., for the application of autonomous devices, we mainly show the online training scheme. Figure 4(b) presents the conductance evolution of ten synapses in the online training process of a single sinusoid wave pattern, randomly chosen from **W**^NN^ and **W**^ZN^. The initial synaptic states are randomly set. As shown, each synapse always tunes itself to force the output to approach the target pattern under applied modulation pulse train at each time step. After hundreds of learning iterations, the weights gradually converge to the stable states. All the joint conductance values shown in Fig. 4(b) are within the range [−3 mS, 3 mS], which is consistent with the modulation range mentioned earlier. For the sake of comparison, the offline training process is also illustrated in Supplementary Fig. S5.

Not limited to single target pattern, the recurrent network is also able to learn and reproduce multiple patterns at the same time. For example, Fig. 4(c) shows the result of learning two target patterns simultaneously, which are composed of two different compositions of sinusoid waves with distinct amplitude ratios. In fact, this ability results from the multiple stable fixed points of the recurrent network with strongly nonlinear dynamics. Driven by two different static control inputs, the network output is able to approach the corresponding fixed points via modulating the internal synaptic weights. As a hardware system, it is necessary to consider the variation of memristor switching characteristics (including variation from device to device and from cycle to cycle in the same device), which is one of the main obstacles to integrate a large-scale memristor array^39^,^40^,^59^,^60^. In order to investigate the system immunity to device variation, the network is simulated using the fuzzy finally trained weights with a dispersion of ±16% at most. As shown in Fig. 4(d), the system reproduces the two different patterns well at a weight dispersion of ±6% despite of the phase error between the actual output and the target pattern. One of the two target patterns can still be reasonably reproduced even at a weight deviation of ±12%, but both fail at ±16%. In general, the network can tolerate the device variation of memristor to a great extent.

### Application for human motor generation

**Figure 5:**
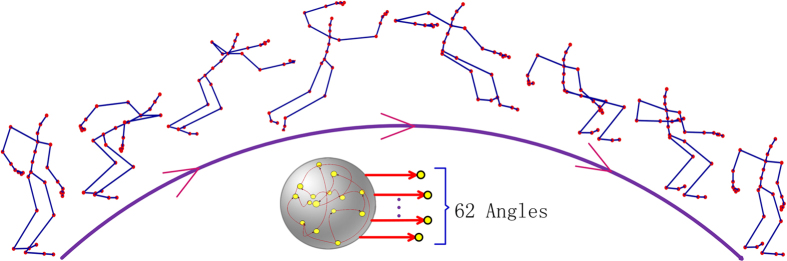
The application of human motor generation. An example of human motor pattern is generated by this system. The trajectory of 62 joint angle datasets from human capture library is learned by assigning 62 readout units. The connected synapses in each subset of the internal neurons are modulated using the error between each actual output and its corresponding ideal joint angle. After training, the network generates the jump motion pattern by itself. For illustration, only eight frames of the whole pattern are chosen to be presented.

To implement complex learning in real world application, we demonstrate a relatively complex example of human motor pattern generation, similar to the learning function in brain motor cortex. We use the captured data of human jump motion from the MOCAP library^61^ to train the recurrent network. The data set contains more than one thousand frames and each frame is comprised of 62 joint angles measured by real human actions^62^. The jump motion as a whole forms an extremely complex spatiotemporal pattern with 62 trajectories. Therefore, 62 readout units are assigned to the network, standing for the spatial coordination of 62 human joints, respectively. The synapses, connecting each subset of the internal neurons, are modulated using the error between each actual output angle and its corresponding target angle. Figure 5 shows the learning results, where only eight frames of the output pattern after training are displayed. It is shown that the proposed bio-plausible recurrent memristive network is able to produce complex motor pattern by the self-adaptation of synapses. This example suggests good potential of the applications of robotics^63^ and motor control in mobile devices.

## Discussion

In summary, the proposed framework successfully bridges the complex learning behaviors of the brain and efficient neuromorphic engineering. This framework, implemented by iron oxide memristor-based synapse, bio-plausible recurrent network and dedicated RLS learning algorithm, is able to produce various complex spatiotemporal patterns after online training. This memristive system also shows a good tolerance to the memristor variation. Such results provide a methodology to explore the complex learning functions of the brain cortex in neuromorphic systems.

The demonstration of complex learning in this paper is very promising in opening up new application space for future intelligent devices. However, to translate this possibility into actuality, it is highly required to extend this work to large-scale memristive systems. One challenging issue is how to build large-scale memristive crossbar and tightly integrate memristors with conventional CMOS circuits. Thus it seems necessary to develop 2-D or 3-D memrsitor integration technology^25^,^64^ and improve the compatibility with CMOS process^65^. It is also desirable that the organization of the hardware system is flexible and scalable. Another challenge is how to map a large-scale functional model into the modular hardware system in an efficient fashion. It is necessary to develop an elaborate mapping compiler, an optimized routing strategy among large amount of neurons, as well as a robust information coding scheme. In addition to recurrent dynamics, the super-complex learning behaviors of the brain also arise from hierarchical network structure and information flow^66^. This can be seen from the success of deep neural networks^3^ in machine learning, which is inspired from the hierarchy of brain computation. As a result, how to efficiently train a hierarchical neural network, especially with recurrent connections, is challenging and worthwhile investigating. In general, the proposed framework and implementation of complex learning in memristive networks lay a foundation for large-scale neuromorphic systems with powerful spatiotemporal learning ability.

## Methods

### Device structure and fabrication

The memristor consists of the iron oxide switching layer sandwiched by two metal electrodes, as shown in Fig. 1(a). The bottom electrode (100 nm TiW and 45 nm Pt) is first deposited in sputter systems on a silicon substrate SiO_2_/Si (1000 nm/525 μm). It is patterned using lift off process with conventional optical lithography. On top of the bottom electrode, the active layer (50 nm iron oxide) is sputtered from an iron oxide compound target at room temperature in Ar ambient. The active layer is then patterned to a 10 μm ×10 μm square using dry etching process. Then a 45 nm-thick SiO_2_ is deposited and a nano hole of 0.25 μm^2^ size is dry-etched under the assist of E-beam lithography to expose the active layer. By using the lift-off processes, the top electrode (45 nm Pt and 100 nm TiW) is patterned finally. Based on X-ray photoelectron spectroscopy (XPS) analysis, the majority compound of active layer is FeO.

### Experimental measurement

The iron oxide memristor is first tested under DC mode on Keithley instrument. A triangle-wave shape DC voltage is applied to the memristor. The voltage is linearly increased from 0 V to 0.65 V, then linearly decreased back to 0 V. The current curve in each sweeping cycle appears a banana-shape like hysteresis loop during both positive sweeping (in the first quadrant) and negative sweeping (in the third quadrant), as shown in Fig. 1(b). As the number of cycles increases, the conductance increases or decreases monotonically and consecutively. During the voltage sweeping, there is no fluctuation or abrupt change in current curves, implying a continuous distribution of resistance states. The iron oxide memristor is also tested under pulse mode on Keithley instrument too. The arbitrary positive pulse and negative positive pulse train are constructed with the amplitude of 1.6 V and −1.6 V, respectively. The pulse width of both pulses is set to 10 μs. During the test, a number of 15 positive pulses are first applied to memristor consecutively, followed by another 15 negative pulses. At the interval of the pulses, the instrument switches to DC mode to read the resistance at 100 mV automatically. In order to achieve a higher precision when modulating the weight, a new method of differential pulse pair modulation has been proposed and experimentally measured, as shown in Supplementary Fig. S2. In this measurement, the write and read manner is similar to above unidirectional pulse modulation. The pulse pair is comprised of a 2 V pulse and a −2 V pulse, with the pulse width of 100 μs. By applying the pulse pair, the minimum step of resistance change can be as small as 0.6 Ω, which indicates that the precision could reach 0.3% at the level of low resistance state.

## Additional Information

**How to cite this article**: Deng, L. *et al*. Complex Learning in Bio-plausible Memristive Networks. *Sci. Rep.* **5**, 10684; doi: 10.1038/srep10684 (2015).

## Supplementary Material

Supplementary Information
