# Supplementary material for: Complex Learning in Bio-plausible Memristive Networks
Source: Sci Rep. 2015 Jun 19;5:10684. doi: 10.1038/srep10684 (PMC4473596; doi:10.1038/srep10684)
Supplement: Supplementary Information [file srep10684-s1.pdf]

Supplementary Information

Complex Learning in Bio-plausible Memristive Networks

Lei Deng*[1,2], Guoqi Li*[1,2], Ning Deng[1,3], Dong Wang[1,2], Ziyang Zhang[1,2],

Wei He[4], Huanglong Li[1,2], Jing Pei[1,2], Luping Shi[1,2]

[1]Center for Brain-Inspired Computing Research (CBICR), Department of Precision Instrument, Tsinghua University, Beijing 100084, China

[2]Optical Memory National Engineering Research Center, Department of Precision Instrument, Tsinghua University, Beijing 100084, China

[3]Institute of Microelectronics, Tsinghua University, Beijing 100084, China

[4]Data Storage Institute, Agency for Science, Technology and Research (A*STAR), 5 Engineering Drive 1, Singapore 117608

Corresponding: lpshi@mail.tsinghua.edu.cn, and peij@mail.tsinghua.edu.cn

*These authors contributed equally to this work.

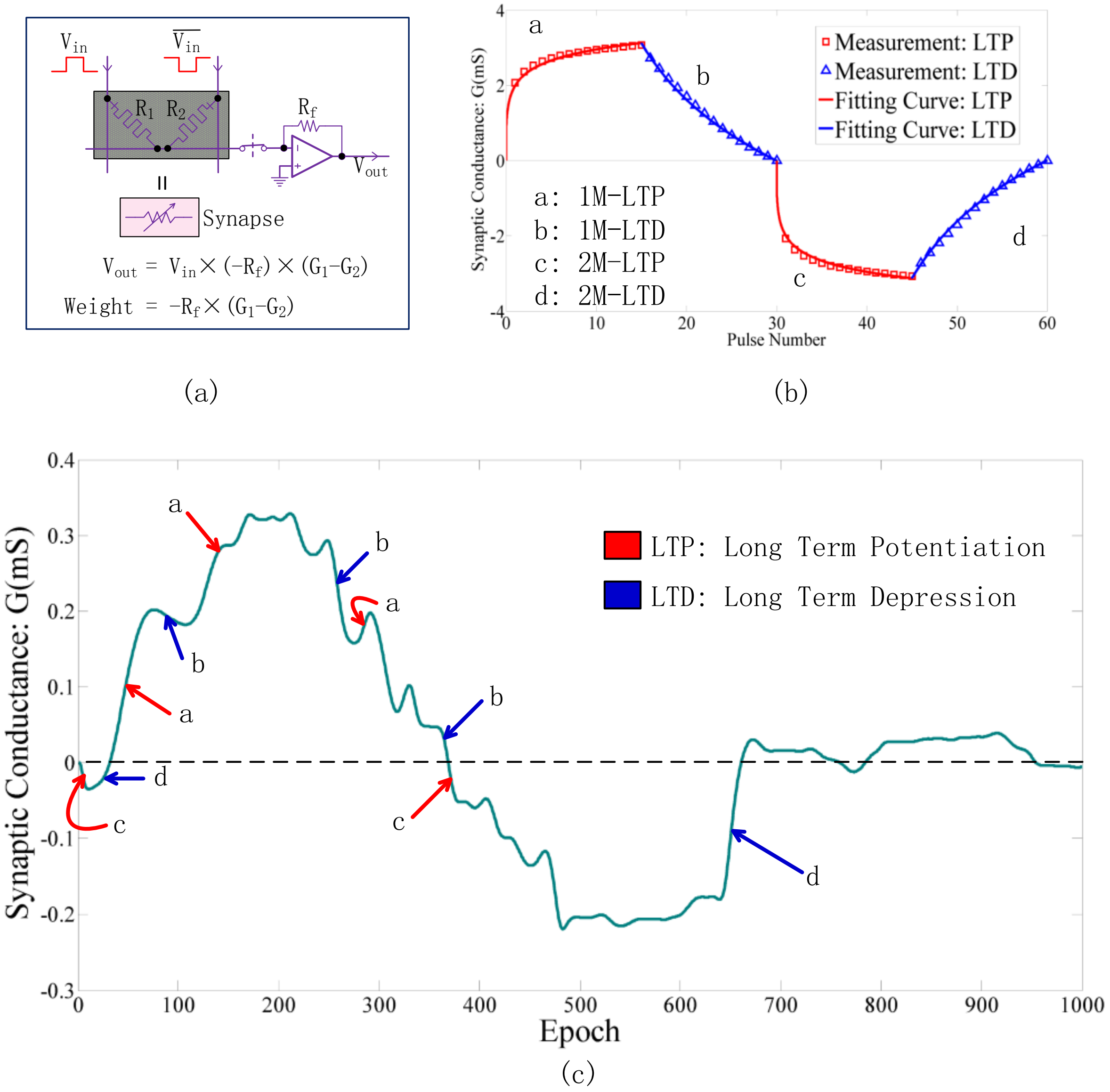

Supplementary Figure S1 | Four cases of weight modulation. (**a**) Schematic of the synapse structure composed of two parallel memristors and one amplifier. The left memristor has positive contribution to the synaptic weight, and the right one with reverse input voltage has negative contribution. (**b**) Illustration of four modulating cases of the two parallel memristors in a synapse. There is only one memristor in the synapse is allowed to change its conductance during the modulation stage, which ensures a unique modulation path to drive the synaptic weight from any initial value to the desired one. The joint conductance of the synapse could be gradually modulated from -3 mS to +3 mS. The definition of modulation cases: a. potentiation of the left memritor (1M-LTP); b. depression of the left memritor (1M-LTD); c. potentiation of the right memristor (2M-LTP); d. depression of the right memritor (2M-LTD). (**c**) An example of the synaptic modulating process when learning. The red arrows denote the potentiation process of one memristor in the synapse, and the blue arrows denote the depression process. Only the typical modulation events in the first 1000 epochs are presented.

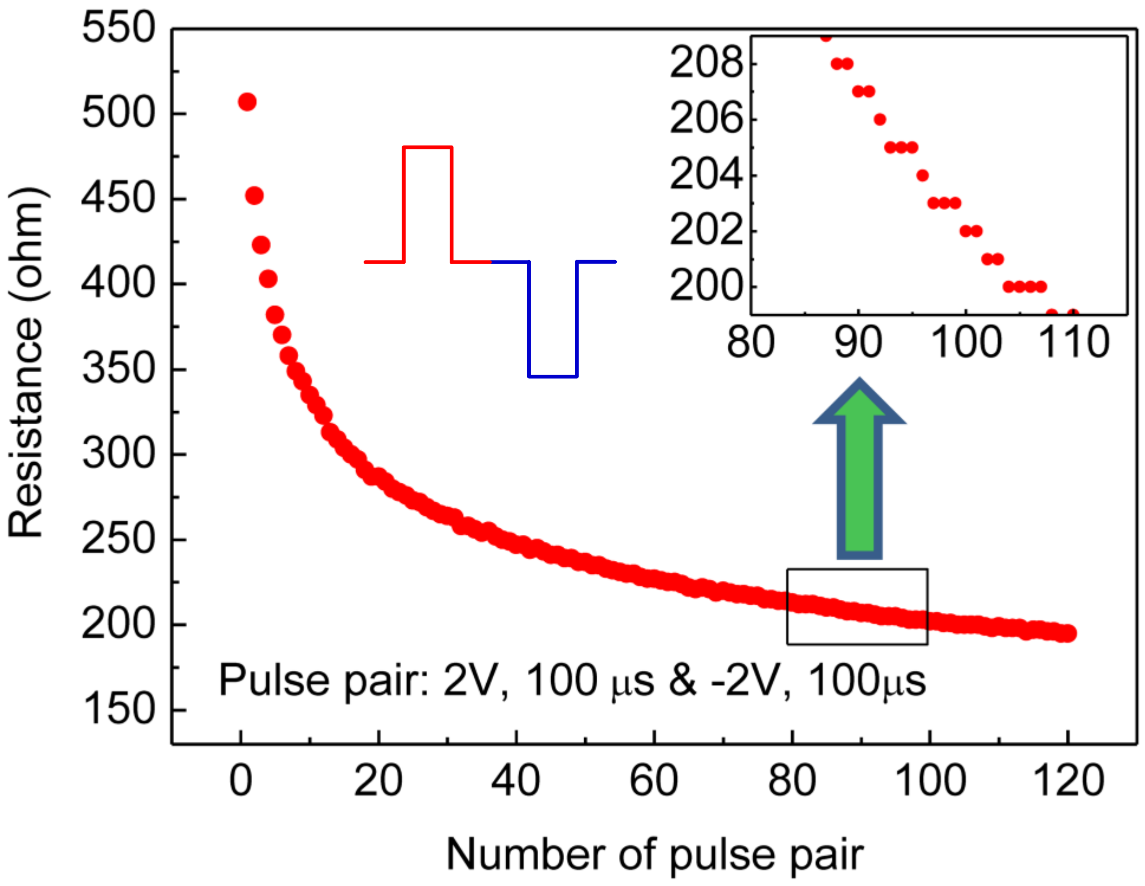

Supplementary Figure S2 | High-precision modulation under differential pulse pair. In order to achieve a higher precision when modulating the weight, a pair of differential pulses consisting of a positive pulse and a negative pulse is used to modulate the memristor resistance to the desired value. In our measurement, the positive pulse has the amplitude of 2V and pulse width of 100 μs. The negative pulse has the same pulse amplitude and width, but different pulse direction. By applying the pulse pair, the resistance of memristor can be tuned in a more precise way instead of the big strike if we just use one side of pulse to adjust the resistance, for instance in the process of Fig. 1(c). As shown inside the inset of this graph, the minimum step of resistance change is as small as 0.6 Ω, which indicates that the relative precision could reach 0.3% at the 200 ohm level of low resistance state. By varying the pulse amplitude and width of pulse pair, a higher accuracy can be further achieved.

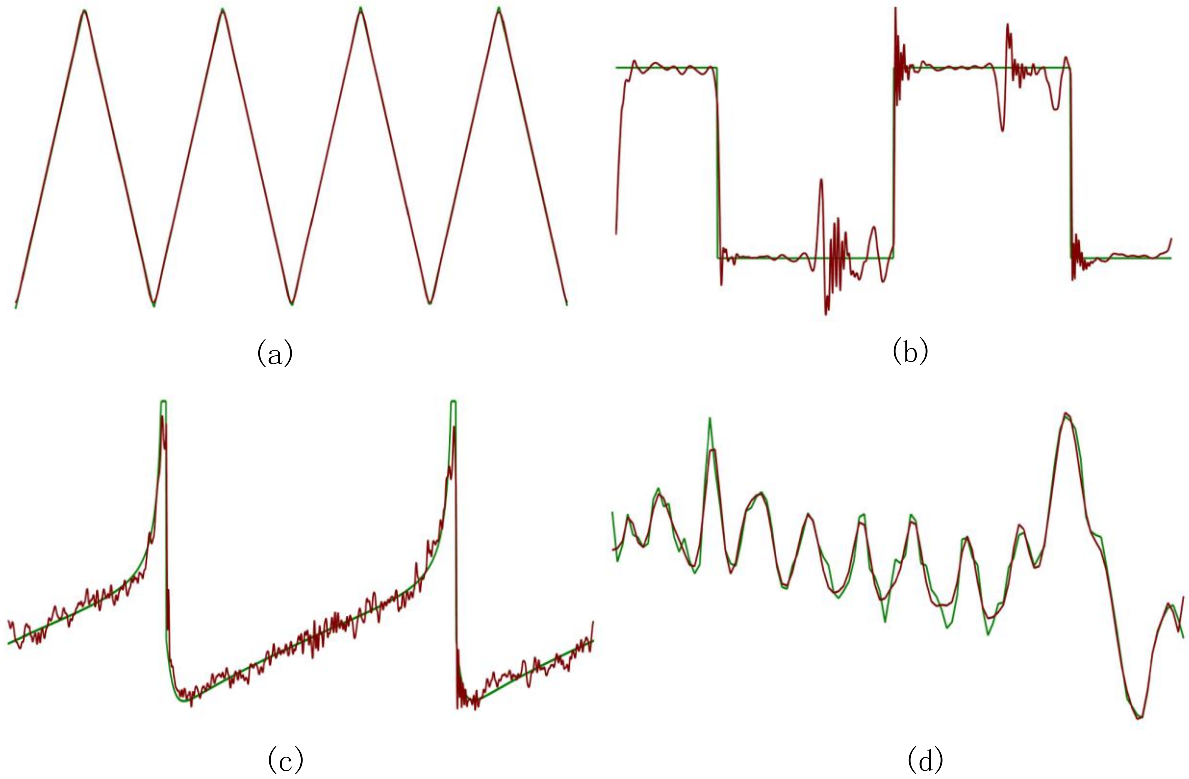

Supplementary Figure S3 caption text follows:

Supplementary Figure S3 | Various timing patterns generation. Besides the common patterns shown in Fig. 4, the system is also trained to produce some more complex patterns in real world even with discontinuous derivative, for example: (**a**) triangular wave; (**b**) square wave; (**c**) spiking activity of Izhikevich neuron [1]; (**d**) a segment of electrocardiogram (ECG) signal. Because of the sharp change of these signals, the output of the network often presents obvious fluctuation; nevertheless, the output has a fairly similar shape to that of the target pattern.

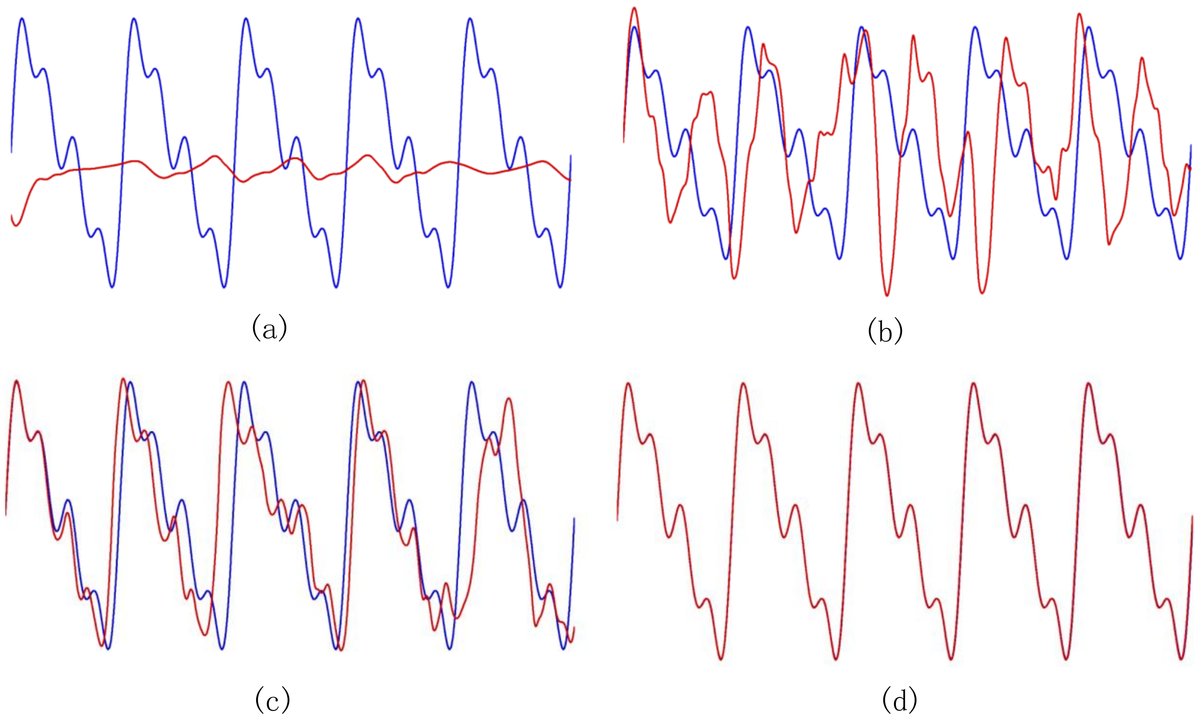

Supplementary Figure S4 | Evolution of pattern generation from chaotic activity. The regular output pattern after training emerges from the chaotic activity of the recurrent neuromorphic network. The red traces denote the autonomous output of this network after training, and the blue traces are the target patterns. It is a typical evolutionary history: (**a**) start-up phase with totally different shapes; (**b**) set-up phase to approach the target shape; (**c**) improvement phase to reduce error; (**d**) final phase with perfect shape and minimum error (the output and the target pattern almost overlap with each other). Because of the much less strict requirement of the initial state in the bio-plausible network, it can tackle better with the memristor variation.

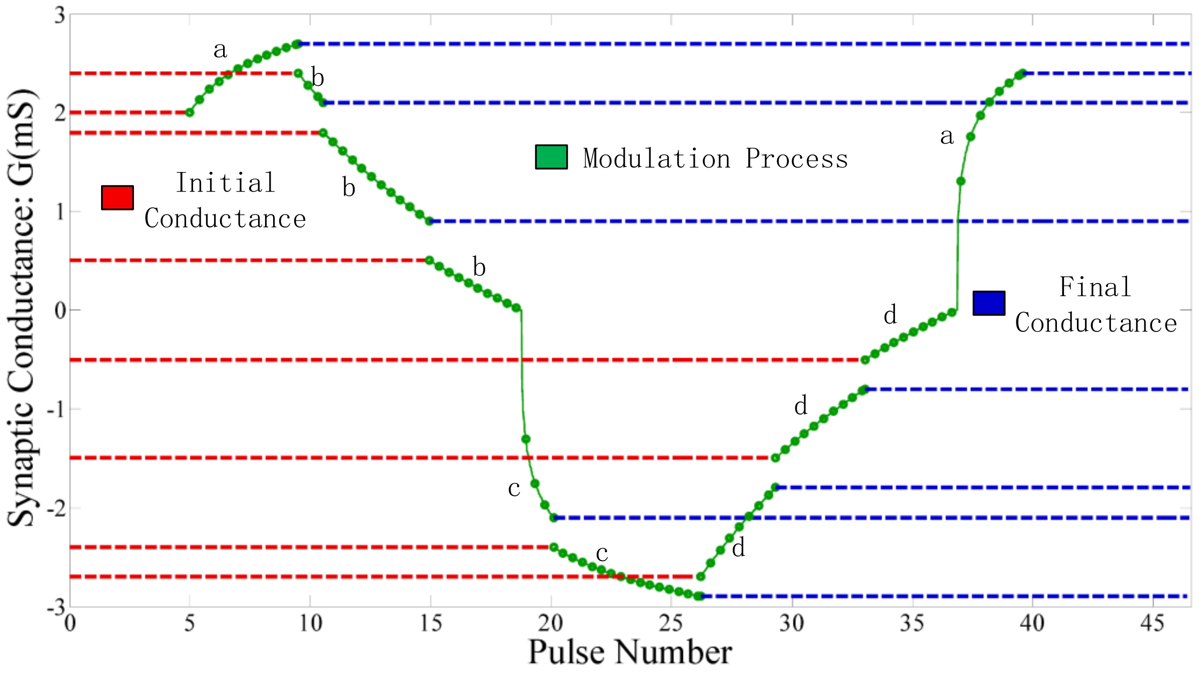

Supplementary Figure S5 | An example of synapse adaptation during offline learning. Different from the numerous iterations of online modulation, the offline training only requires to import the finally optimized synaptic weights into the network after software-based parameter calculation. The red curves denote the artificially generated conductance of several synapses, the blue curves are the finally optimized synaptic conductance values. The figure is mainly aimed to capture the once modulation process during offline learning, so the green traces directly leverage the fitting curve in Supplementary Figure S1(b). In order to achieve a clear layout without overlap between two modulation processes, the eight synapses are modulated sequentially one by one. If we introduce the feedback algorithm to precisely modulate the synapses, the green modulation traces would show obvious fluctuation because of the alternating switching between LTP direction and LTD direction [2].

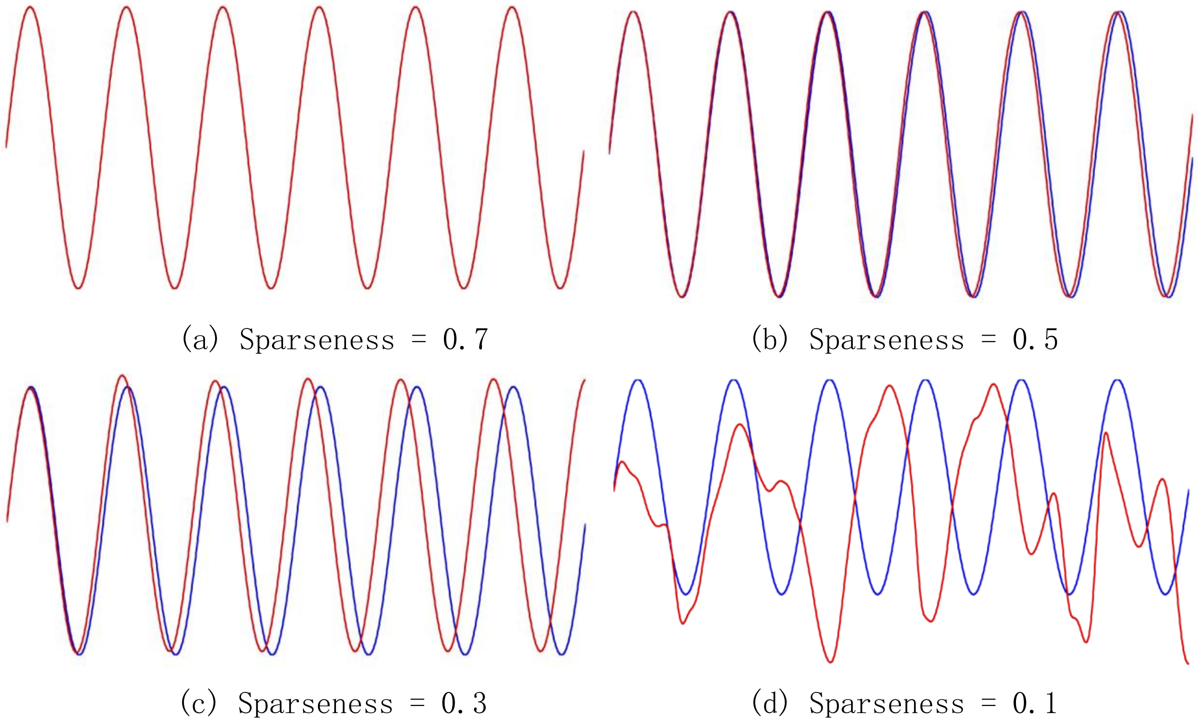

Supplementary Figure S6 | Analysis of building sparse networks. (**a**) The output error still remains very small even at the sparseness of 0.7. (**b**) & (**c**) The output pattern starts to present phase error but still keeps the right shape at the sparseness of 0.5 and 0.3. (**d**) Because of the abrupt reduction of number of the active synapses, the ability to learn spatiotemporal pattern finally collapses at the sparseness of 0.1. A sparse network is very helpful to reduce the hardware overhead, especially in a large-scale system.

Device mechanism and behavior model

In order to obtain analog resistance states of the iron oxide-based memristor, a pre-forming process and an initial reset are required before operation. The resistive switching is believed to be due to the growth and rupture of the conducting filament, which is driven by the oxygen vacancy migration. The intermediate resistance states and consequently the LTP or LTD behaviors might correlate to factors like the width of the depletion gap, the diameter of the filament, the number of filaments, etc. Our device shows a good analog behavior due to the optimized oxygen vacancy drift and diffusion fluxes, that is, both of the fluxes are of moderate strength [3], which are in turn subject to the small hopping distance of electrons from one oxygen vacancy to another. Further study is needed to justify the argument of small hopping distance in our FeO layer. The reasonably good thermal conductivity of Pt top electrode also contributes to the good analog property that fast dissipation of joule heating through the TE decreases both drift and diffusion fluxes, resulting in gradual resistive switching.

To support the simulation, we introduce a phenomenological model [4] to emulate the behaviour of the memristive device:

$$\frac{dG}{dt} = \pm A e^{\left(-B\frac{G-G_{min}}{G_{max}-G_{min}}\right)}$$

where G, G_{min}, G_{max} are the conductance, minimal conductance and maximal conductance of memristor, respectively. A and B are two fitting parameters. The plus sign before A indicates the LTP process and the minus sign is the LTD process. The simulation results are shown in Supplementary Figure S1(b).

High-precision modulation of memristor conductance

To build the memristor-based neuromorphic computing system, the most challenging work is to precisely modulate the memristive states. Owing to the proposed synapse structure shown in Supplementary Figure S1(a), we could achieve a wider weight range compared with that of the conductance-based synapse, by matching the feedback resistance R_f of the inverting amplifier. Theoretically, it’s reasonable to use the continuous curve in Supplementary Figure S1(b) obtained from the device measurements to support the simulation model [4, 5]. The illustration of online weight adaptation is also presented in Supplementary Figure S1(c). We tried to achieve a higher tuning precision in contrast to the crude modulation in Fig. 1(c). As shown in Supplementary Figure S2, we propose a differential pulse pair scheme to tune the memristive states. By choosing the amplitude and width of the pulse pair, the relative precision could reach 0.3%. However, these methods are still based on open-loop modulation, which is tough to tackle the device variation in real hardware system, including variation from device to

device and cycle to cycle. A natural way to deal with the issue is to utilize a closed-loop scheme or feedback algorithm [2, 6]. In the feedback modulation, complex pulse train (often varying amplitude and width) and repeatedly write/read operations are required. Nevertheless, in the sense of synaptic modulation, our bio-plausible recurrent network and learning algorithm are feasible in memritor-based neuromorphic hardware system, especially with the development of the crossbar and 3-D memristor integration technology [7, 8].

Offline and online training scheme

By introducing the classical RLS algorithm in control theory, the recurrent network is trained to produce various complex spatiotemporal patterns in Supplementary Figure S3, including the bio-plausible neuronal spiking activity and ECG signal. In fact, these complex patterns emerge from the internal chaotic activities of the network, as shown in Supplementary Figure S4, which is crucial to the timing coding of the brain [9, 10]. In our work, we fix g at 1.5 to remain the initial network in a chaotic dynamic regime [11]. In order to produce specific target pattern, the network must be trained to reach the corresponding stable state. There are two schemes to train a memristive network, online training and offline training [2, 12]. Online training requires a rapid weight modulation at each time step and needs longer training time; while offline training only needs a final importation of the predefined parameters, as illustrated in Supplementary Figure S5. In this sense, the offline modulation is faster, and without time limitation between two iterations, we can further introduce the feedback tuning mentioned earlier to achieve a higher precision. However, offline training heavily depends on the software-based precursor system for parameter computing so that it is hard to be embedded into portable devices. In our implementation, we prefer the online scheme for the future practical application. In this context, the implementation should take into account the limited time interval to modulate the memristive states at each time step. In principle, fortunately, there is no need to consider this issue because the switching speed of memristor could be $\sim 10^3 - \sim 10^6$ times faster than that of the brain operation. Accordingly, we can update the weights one by one. Whereas, the number of synapses will increase rapidly when the network scales up because of the crossbar architecture. As a result, the burden of the pulse modulator will be enormously heavy. One solution is to adopt parallel programming method in the memristor array. Another solution is to construct a sparsely connected network consisting of much fewer active synapses to alleviate the modulation burden. As shown in Supplementary Figure S6, our recurrent network is able to produce the right pattern even at the sparseness of 0.3. This promises to support the large-scale neuromorphic networks for online learning in the future.

References

[1] Izhikevich, E. M. Simple model of spiking neurons. *IEEE Trans. on neural network.* **14**, 1569-1572 (2003).

[2] Alibart, F., Zamanidoost, E. & Strukov, D. B. Pattern classification by memristive crossbar circuits using ex situ and in situ training. *Nat. Commun.* **4**, 2072 (2013).

[3] Kim, S., Choi, S. H. & Lu W. Comprehensive physical model of dynamic resistive switching in an oxide memristor. *ACS nano* **8**, 2369-2376 (2014).

[4] Suri, M. et al. Physical aspects of low power synapses based on phase change memory devices. *J. Appl. Phys.* **112**, 054904 (2012).

[5] Jang, J. W. et al. ReRAM-based synaptic device for neuromorphic computing. *IEEE Int. Symposium on Circuits and Systems* **2014**, 1054-1057 (2014).

[6] Alibart, F. et al. High precision tuning of state for memristive devices by adaptable variation-tolerant algorithm. *Nanotechnology* **23**, 075201 (2012).

[7] Park, S. et al. Rram-based synapse for neuromorphic system with pattern recognition function. *IEEE Int. Electron Devices Meet.* **2012**, 10.2. 1-10.2. 4 (2012).

[8] Yu, S. et al. HfOx-based vertical resistive switching random access memory suitable for bit-cost-effective three-dimensional cross-point architecture. *ACS Nano* **7**, 2320-2325 (2013).

[9] van Vreeswijk, C. & Sompolinsky, H. Chaos in neuronal networks with balanced excitatory and inhibitory activity. *Science* **274**, 1724-1726 (1996).

[10] Wallace, E., Maei, H. R. & Latham, P. E. Randomly connected networks have short temporal memory. *Neural Comput.* **25**, 1408-1439 (2013).

[11] Sussillo, D. & Abbott, L. F. Generating coherent patterns of activity from chaotic neural networks. *Neuron* **63**, 544-557 (2009).

[12] Merolla, P. et al. A million spiking-neuron integrated circuit with a scalable communication network and interface. *Science* **345**, 668-673 (2014).
